# Malignancy risk estimation of screen-detected nodules at baseline CT: comparison of the PanCan model, Lung-RADS and NCCN guidelines

**DOI:** 10.1007/s00330-017-4767-2

**Published:** 2017-03-14

**Authors:** Sarah J. van Riel, Francesco Ciompi, Colin Jacobs, Mathilde M. Winkler Wille, Ernst Th. Scholten, Matiullah Naqibullah, Stephen Lam, Mathias Prokop, Cornelia Schaefer-Prokop, Bram van Ginneken

**Affiliations:** 1 Department of Radiology and Nuclear Medicine, Radboud University Nijmegen Medical Center, Nijmegen, The Netherlands; 2 Department of Pulmonology Gentofte Hospital, University of Copenhagen, Hellerup, Denmark; 3 Department of Integrative Oncology, British Columbia Cancer Agency, Vancouver, British Columbia Canada; 4 Department of Radiology, Meander Medical Center, Amersfoort, The Netherlands

**Keywords:** Lung cancer screening, Diagnostic imaging, Computer tomography, Solitary pulmonary nodule, Risk

## Abstract

**Objectives:**

To compare the PanCan model, Lung-RADS and the 1.2016 National Comprehensive Cancer Network (NCCN) guidelines for discriminating malignant from benign pulmonary nodules on baseline screening CT scans and the impact diameter measurement methods have on performances.

**Methods:**

From the Danish Lung Cancer Screening Trial database, 64 CTs with malignant nodules and 549 baseline CTs with benign nodules were included. Performance of the systems was evaluated applying the system's original diameter definitions: D^longest-C^ (PanCan), D^meanAxial^ (NCCN), both obtained from axial sections, and D^mean3D^ (Lung-RADS). Subsequently all diameter definitions were applied uniformly to all systems. Areas under the ROC curves (AUC) were used to evaluate risk discrimination.

**Results:**

PanCan performed superiorly to Lung-RADS and NCCN (AUC 0.874 vs. 0.813, p = 0.003; 0.874 vs. 0.836, p = 0.010), using the original diameter specifications. When uniformly applying D^longest-C^, D^mean3D^ and D^meanAxial^, PanCan remained superior to Lung-RADS (p < 0.001 – p = 0.001) and NCCN (p < 0.001 – p = 0.016). Diameter definition significantly influenced NCCN’s performance with D^longest-C^ being the worst (D^longest-C^ vs. D^mean3D^, p = 0.005; D^longest-C^ vs. D^meanAxial^, p = 0.016).

**Conclusions:**

Without follow-up information, the PanCan model performs significantly superiorly to Lung-RADS and the 1.2016 NCCN guidelines for discriminating benign from malignant nodules. The NCCN guidelines are most sensitive to nodule size definition.

***Key Points*:**

• *PanCan model outperforms Lung*-*RADS and 1.2016 NCCN guidelines in identifying malignant pulmonary nodules*.

• *Nodule size definition had no significant impact on Lung*-*RADS and PanCan model*.

• *1.2016 NCCN guidelines were significantly superior when using mean diameter to longest diameter*.

• *Longest diameter achieved lowest performance for all models*.

• *Mean diameter performed equivalently when derived from axial sections and from volumetry*.

## Introduction

The cost-effectiveness of a lung cancer screening programme is influenced by the lung nodule management protocol [1]. Differentiating high-risk nodules from low-risk nodules based on nodule characteristics remains a difficult task [2, 3]. To aid radiologists in recommending the most appropriate follow-up procedure, several categorical management protocols and scoring systems have been published recently. In 2014, the American College of Radiology (ACR) published version 1.0 of the Lung-RADS Assessment Categories to standardize the CT lung screening reporting and management recommendations [4]. Both Lung-RADS and the 1.2016 National Comprehensive Cancer Network (NCCN) base their nodule management on nodule type, size and nodule growth over time [5]. Their category definitions and associated management recommendations have been determined empirically based on previous publications and clinical experience, resulting in slight variations across systems.

In 2013, the PanCan prediction models were published [6], estimating the probability of cancer in pulmonary nodules detected on first-screening CT scans. The parameters of these models were mathematically derived based on screening data. In addition to nodule-related aspects such as size, type or location, they take subject characteristics into account to compute a nodule risk index on a continuous scale. Validation of these models was performed using data from chemoprevention trials of the British Columbia Cancer Agency (BCCA) [6], and independently of that using the Danish Lung Cancer Screening Trial (DLCST) [7], revealing areas under the receiver operating characteristic (ROC) curve (AUC) ranging from 0.940 for the first to 0.834 for the latter.

The nodule management strategy has a large impact on screening programmes, especially when large databases need to be analysed. Since it remains unclear which approach works best to determine the subgroup of screen-detected nodules that require more intense work-up, we were interested in the performance differences between these three strategies.

The purpose of this study was to compare the performance of the PanCan model, Lung-RADS and the 1.2016 NCCN guidelines to differentiate the same set of malignant from benign screen-detected pulmonary nodules and to determine the impact of different diameter measurement methods on the model’s performances.

## Methods

### Materials

All study cases in this retrospective study were derived from the DLCST. Details regarding the DLCST protocol have been reported previously [8]. Approval by the DLCST ethics committee as well as informed consent from all participants were received.

### Data selection

We considered nodules annotated by at least one of the two screening radiologists. From the baseline annotations, two groups were defined: (i) nodules that were found to be cancer and (ii) nodules considered benign within the follow-up of approximately 9 years. In total, 70 primary lung cancers were found in 64 participants. For the malignant nodules, the scan on which the malignant nodule was annotated for the first time by at least one screening radiologist was included in the study. As a consequence, for 29 cancers in 27 participants, the scan included in this study did not represent the baseline CT (T0) scan. For eight of these participants the T0 scans with annotated benign nodules were excluded, in order to have only one CT scan input per participant. All other benign nodules annotated at T0 by a screening radiologist were included. In total, the dataset consisted of 930 nodules in 613 participants: 70 malignant nodules in 64 participants and 860 benign nodules in 549 participants.

### CT acquisition

All CT scans were performed using a multi-detector CT scanner (16-row Philips Mx 8000, Philips Medical Systems, Eindhoven, The Netherlands), with a low-dose protocol of 120 kV and 40 mAs, and reconstructed in thin sections (1 mm) [8].

### Quantitative nodule assessment

All nodules were semi-automatically segmented to assess the longest and perpendicular diameter on axial sections, the mean diameter based on volumetric information of the total nodule and, if present, of the solid component (CIRRUS Observer, Diagnostic Image Analysis Group, RadboudUMC, Nijmegen, The Netherlands). The mean diameter based on volumetry refers to the diameter of a sphere that is adapted as close as possible to the nodule extension in all three dimensions.

### PanCan prediction model

We used the full model including spiculation (2b), which will be referred to as the PanCan model throughout the text to calculate the nodule risk index on a continuous scale for each nodule [6]. The PanCan model considers the parameters age, sex, family history of lung cancer, emphysema, nodule size, nodule type, nodule location, additional nodule count per scan and spiculation. Based on the study by McWilliams et al. [6], the *longest diameter* on axial sections was used as the definition of nodule size, derived from computerized semi-automated volumetric segmentations (D^longest-C^). Manually measured diameters were also available from the DLCST database (D^longest-M^). The parameters nodule type (solid, part-solid, pure ground-glass or perifissural nodule (PFN)), presence of emphysema, spiculation and nodule calcification were scored according to the PanCan model definitions by an experienced radiologist (E.Th.S), who was involved in the readings of the NELSON trial [9]. All other parameters were available from the DLCST database. Completely calcified and perifissural nodules were excluded in the PanCan model and therefore given a nodule risk index of 0% in our study. Although the PanCan model outcome uses a continuous scale in the range of 0–100%, cut-off points of the PanCan model's nodule risk index have been published to serve as a framework to guide clinical investigators, consisting of four categories [10].

### Lung-RADS assessment categories

Lung-RADS consists of five categories, which are based on nodule type, size and growth. Since we did not include follow-up information, we did not consider growth in our study. In the original publication, nodule size was defined as *the average diameter*, rounded to the nearest whole number. We therefore used *the 3D mean diameter* derived from semi-automated volumetric segmentations (D^mean3D^). All nodules were assigned to one of the categories as proposed by the ACR [4].

### National Comprehensive Cancer Network guidelines

The NCCN developed guidelines for management of screen-detected pulmonary nodules, based on nodule type, size and nodule growth. Nodule size was defined in the guidelines as the *mean of the longest diameter and the perpendicular diameter* on axial sections (D^meanAxial^). In this study we used the NCCN guidelines published in 2015 (version 1.2016). All nodules in our study were assigned to one of six categories as proposed by the guidelines [5].

The criteria for the categories of the scoring systems described above are specified in Table 1.

**Table 1:** Overview of nodule management scoring system categories and corresponding criteria

Scoring system	Category	Criteria	Management
PanCan	1	Normal finding, nodule risk index <1.5%	Biennial LDCT screening
	2	Low-risk of malignancy: nodule risk index 1.5% – <6%	Annual LDCT screening
	3	Moderate risk of malignancy: nodule risk index 6% – <30%	3-month LDCT
	4	High-risk of malignancy: nodule risk index ≥30%	Direct referral
Lung-RADS (version 1.0)	1	No nodules, or nodules with complete, central, popcorn, or concentric rings of calcification, fat containing nodules	Annual LDCT screening
	2	Solid nodules < 6 mm Part-solid nodules <6 mm in total diameter Pure ground-glass nodules <20 mm	Annual LDCT screening
	3	Solid nodules ≥ 6 – <8 mm Part-solid nodules ≥ 6 mm in total diameter with solid component < 6 mm Pure ground-glass nodules ≥20 mm	6-month LDCT
	4A	Solid nodules ≥ 8 – < 15 mm Part-solid nodules ≥6 mm with solid component ≥6 – <8 mm	3-month LDCT; PET/CT
	4B	Solid nodules ≥ 15 mm Part-solid nodules with a solid component ≥ 8 mm	Chest CT with/without contrast, PET/CT and/or tissue sampling
NCCN (version 1.2016)	1	Solid nodule or part-solid nodule < 6 mm	Annual LDCT for 2 years
	2	Pure ground-glass nodule ≤ 5 mm Multiple pure ground-glass nodules ≤ 5 mm	LDCT in 12 months
	3	Pure ground-glass nodule > 5-10 mm Multiple pure ground-glass nodules with a diameter >5 mm without a dominant lesion	LDCT in 6 months
	4	Pure ground-glass nodule > 10 mm Multiple pure ground-glass nodules with a dominant nodule(s) with a solid component	LDCT in 3–6 months
	5	Solid nodule or part-solid nodule 6–8 mm	LDCT in 3 months
	6	Solid nodule or part-solid nodule > 8 mm	Consider PET/CT

*LDCT* low-dose CT

### Statistical analysis

Per participant, the nodule with the highest nodule risk index or category (risk-dominant nodule) was determined for the two categorical scoring systems and the PanCan model independently. Subsequently, based on the individual performance of the scoring system, different nodules could be determined as being the risk-dominant nodule for the three systems. In participants with multiple risk-dominant nodules for a scoring system, only one risk-dominant nodule was randomly selected per system. A ROC analysis was performed to determine the discriminative power of the PanCan model, Lung-RADS and the 1.2016 NCCN guidelines. Because no follow-up information was included, our analysis is confined to how well PanCan model category 4, Lung-RADS category 4A and 4B, and the 1.2016 NCCN guidelines category 6 predict malignancy. AUC values served as indicators of performance and were calculated for all three scoring systems using MedCalc software package (MedCalc version 16.4.3, https://www.medcalc.org).

To assess differences between the three systems, first the performances were determined applying the system's individual nodule size definition as stated in the publications.

For the PanCan model calculations were done twice: using the manually measured longest diameter available from the DLCST database (D^longest-M^) and using the longest diameter derived from the computerized semi-automatic software segmentations (D^longest-C^).

To assess the susceptibility of the systems to nodule size definition, performances were subsequently determined by applying uniformly the same nodule size definition (D^longest-C^, D^mean3D^ and D^meanAxial^, respectively).

Performances were compared pairewise using the DeLong method [11]. Bonferroni correction was applied for the comparison between the three systems, resulting in a statistically significant difference at p <0.017.

Differences in dataset characteristics were compared using chi-square analysis for categorical data and unpaired t-test analysis for continuous data in SPSS 20.0 (SPSS Inc., Chicago, IL, USA). Statistically significant differences were defined at p <0.05.

## Results

**Fig. 1:**
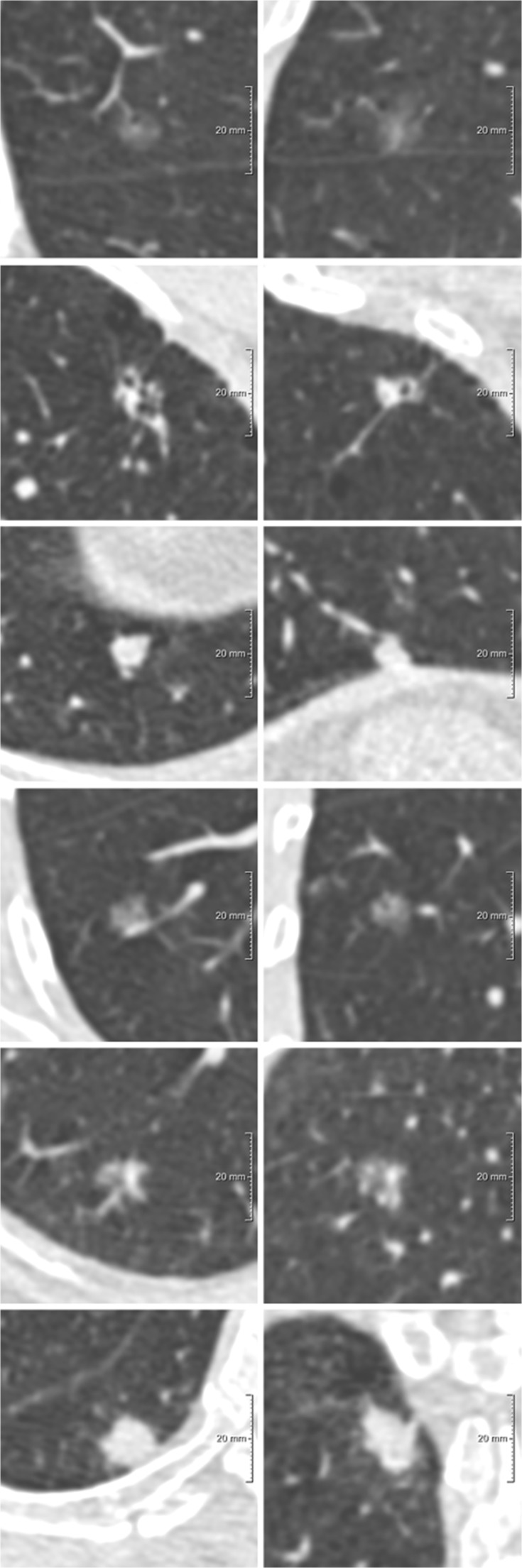
Examples of nodules uniformly considered as the most suspicious nodule per participant by all three systems. Each row depicts a nodule, displayed in the axial (left) and coronal (right) plane and centered in the images with a field of view of 60 x 60 mm. (**A**) *Benign* pure ground-glass nodule, D^longest-C^ 10.0 mm, D^mean3D^ 9.4 mm, D^meanAxial^ 8.8 mm, PanCan model nodule index score of 5.5%, Lung-RADS category 2 and NCCN category 3; (**B**) *Benign* part-solid nodule, D^longest-C^ total nodule 9.4 mm and solid component 7.2 mm, D^mean3D^ total nodule 8.5 mm and solid component 6.2 mm, D^meanAxial^ total nodule 8.8 mm and solid component 6.0 mm, PanCan model nodule index score of 10.0%, Lung-RADS category 4A and NCCN category 6; (**C**) *Benign* solid nodule, D^longest-C^ 10.5 mm, D^mean3D^ 8.4 mm, D^meanAxial^ 9.1 mm, PanCan model nodule index score of 4.4%, Lung-RADS category 4A and NCCN category 6. (**D**) *Malignant* pure ground-glass nodule, D^longest-C^ 12.4 mm, D^mean3D^ 9.8 mm, D^meanAxial^ 11.7 mm, PanCan model nodule index score of 8.4%, Lung-RADS category 2 and NCCN category 4; (**E**) *Malignant* part-solid nodule, D^longest-C^ total nodule 17.4 mm and solid component 7.1 mm, D^mean3D^ total nodule 14.7 mm and solid component 5.2 mm, D^meanAxial^ total nodule 15.7 mm and solid component 6.3 mm, PanCan model nodule index score of 22.2%, Lung-RADS category 3 and NCCN category 6; **F**: *Malignant* solid nodule, D^longest-C^ 15.2 mm, D^mean3D^ 13.5 mm, D^meanAxial^ 13.2 mm, PanCan model nodule index score of 18.1%, Lung-RADS category 4A and, NCCN category 6

**Fig. 2:**
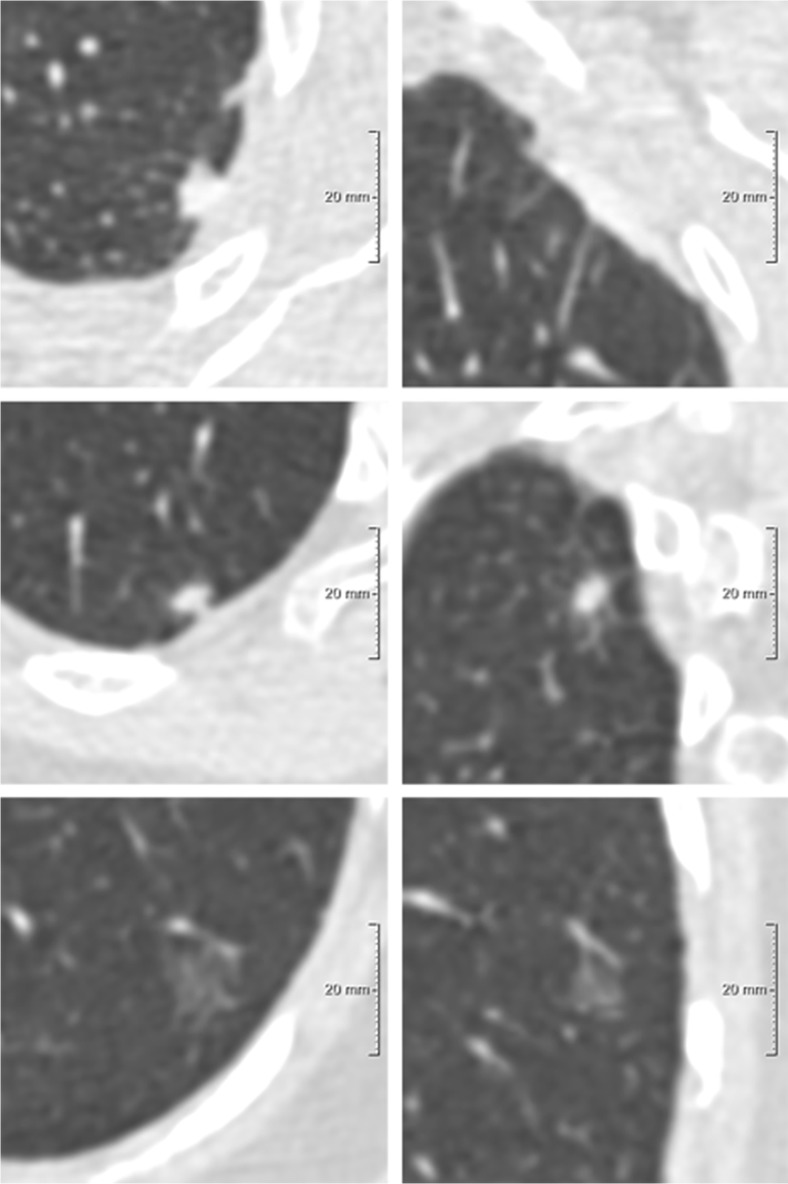
Example of one participant in which three different nodules were considered as the risk-dominant lesion. Each row depicts a nodule, displayed in the axial (left) and coronal (right) plane and centered in the images with a field of view of 60 x 60 mm. (**A**) Solid *benign* nodule, D^meanAxial^ 6.1 mm, risk-dominant nodule for the NCCN guidelines with category 5; (**B**) Solid *benign* nodule, D^mean3D^ 8.7 mm, risk-dominant nodule for Lung-RADS with category 4A; (**C**) Pure ground-glass *benign* nodule, D^longest-C^ 14.3 mm, risk-dominant nodule for the PanCan model with nodule risk index of 0.14%

**Fig. 3:**
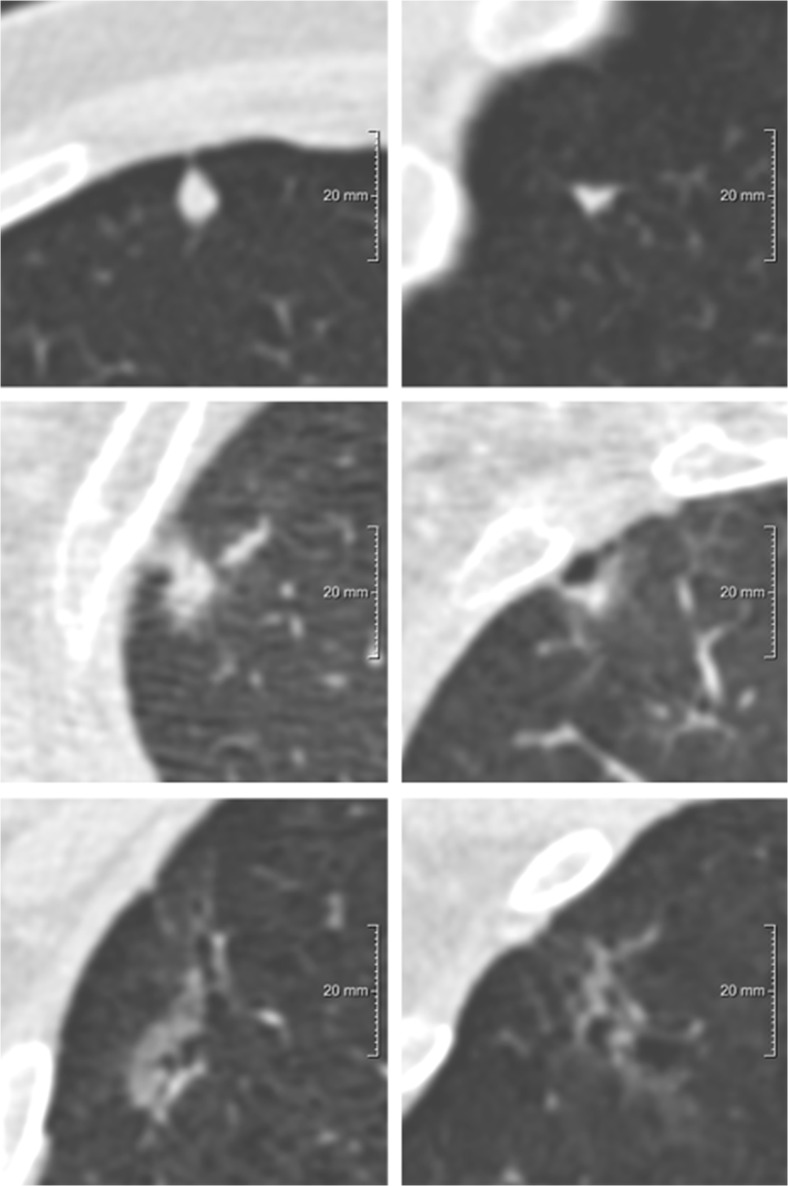
Examples of nodules with variation between the longest diameter, mean diameter based on volumetry, and mean of longest and perpendicular diameter. Each row depicts a nodule, displayed in the axial (left) and coronal (right) plane and centered in the images with a field of view of 60 x 60 mm. (**A**) *Benign* solid nodule with D^longest-C^ 7.8 mm, D^mean3D^ 5.3 mm, D^meanAxial^ 6.3 mm; (**B**) *Malignant* part-solid nodule with D^longest-C^ of total nodule 16.6 mm and of solid component 13.3 mm, D^mean3D^ of total nodule 12.3 mm and of solid component 7.4 mm, D^meanAxial^ of total nodule 15.0 mm and of solid component 10.6 mm; (**C**) *Benign* pure ground-glass nodule with D^longest-C^ 20.6 mm, D^mean3D^ 13.0 mm, D^meanAxial^ 17.0 mm

For 497 participants (497/613, 81%), the three systems identified the same nodule as risk-dominant. In 116 participants (116/613, 19%), the three systems identified different risk-dominant nodules. Figure 1 illustrates nodules considered as being risk-dominant by all systems, while Fig. 2 shows an example of three nodules identified as the risk-dominant nodule by the three systems in a single participant. Figure 3 illustrates examples of nodules where the risk estimation depended on the applied nodule size definition.

Table 2 shows the demographics of the included participants and risk-dominant nodules. Statistically significant differences between the malignant and benign nodules were seen for nodule size (D^longest-C^, D^mean3D^ and D^meanAxial^, all p <0.001), nodule type (p <0.001), perifissural nodule type (p = 0.017), the presence of spiculation (p <0.001), calcification (p = 0.001) or family history of lung cancer (p = 0.043) and nodule count (p = 0.017). The median time between the CT scan used in this study and the date of lung cancer diagnosis was 13.9 months (range 0.3–84.8 months, average 23.2 months)

**Table 2:** Demographics of participants and characteristics of risk-dominant nodules

Parameter	Cancers*	Benign nodules *	Total*	P value**
Number	65	675	740	
Participants	64	549	613	
Age in years	61 (52–75)	58 (50–71)	58 (50–75)	0.655
Sex Male Female	35 (55%) 29 (45%)	292 (53%) 257 (47%)	327 (53%) 286 (47%)	0.820
Family history of lung cancer	17 (27%)	90 (16%)	107 (17%)	**0.043**
Emphysema	47 (73%)	367 (67%)	414 (68%)	0.287
Nodule size in mm: D^longest-C^	Median: 13.9 16.5 (3.3–124.8)	Median: 6.1 7.6 (1.6–104.4)	8.4 (1.6–124.8)	<**0.001**
Nodule size in mm: D^mean3D^	Median: 10.2 12.5 (2.7–84.8)	Median: 4.9 5.8 (1.3–62.3)	6.4 (1.3–84.8)	<**0.001**
Nodule size in mm: D^meanAxial^	Median: 12.1 13.8 (2.9–95.0)	Median: 5.4 6.4 (1.2–95.5)	7.1 (1.2–95.5)	<**0.001**
Nodule type Solid Part-solid Non-solid	43 (66%) 15 (23%) 7 (11%)	590 (87%) 19 (3%) 66 (10%)	633 (85%) 34 (5%) 73 (10%)	<**0.001**
Perifissural	0 (0%)	55 (8%)	55 (7%)	**0.017**
Calcified	0 (0%)	96 (14%)	96 (13%)	**0.001**
Nodule count	0.3 (0–4)	0.5 (0–5)	0.5 (0–5)	**0.017**
Nodule location Upper lobe	38 (58%)	327 (48%)	365 (49%)	0.123
Spiculation	18 (28%)	10 (1%)	28 (4%)	<**0.001**

* Percentages or ranges are in parentheses

** P-value for benign nodules versus cancers. A p-value < 0.05 indicates a significant difference. Family history of lung cancer pertained to parents or siblings. Presence of emphysema was dichotomous and not corresponding to the degree of emphysema. Nodule size was measured as the longest diameter. Nodule count pertained to the number of additional nodules in the scan. Spiculation was defined as reticular markings of tissue density with elements of circular symmetry centered around a nodule

*D* ^*longest*-*C*^ longest diameter on axial sections, derived from computerized semi-automated segmentations, *D* ^*mean3D*^ mean diameter based on volumetric information, derived from computerized semi-automated segmentations, *D* ^*meanAxial*^ mean of longest and perpendicular diameter on axial sections, derived from computerized semi-automated segmentations

### Performance of the three scoring systems

Using the nodule size definitions as described in the original publications, the PanCan model performed best (AUC 0.874, using D^longest-C^), followed by the NCCN guidelines (0.836) and Lung-RADS (0.813). Statistically significant differences occurred between PanCan and Lung-RADS (p = 0.003) and between PanCan and the NCCN guidelines (p = 0.010).

**Fig. 4:**
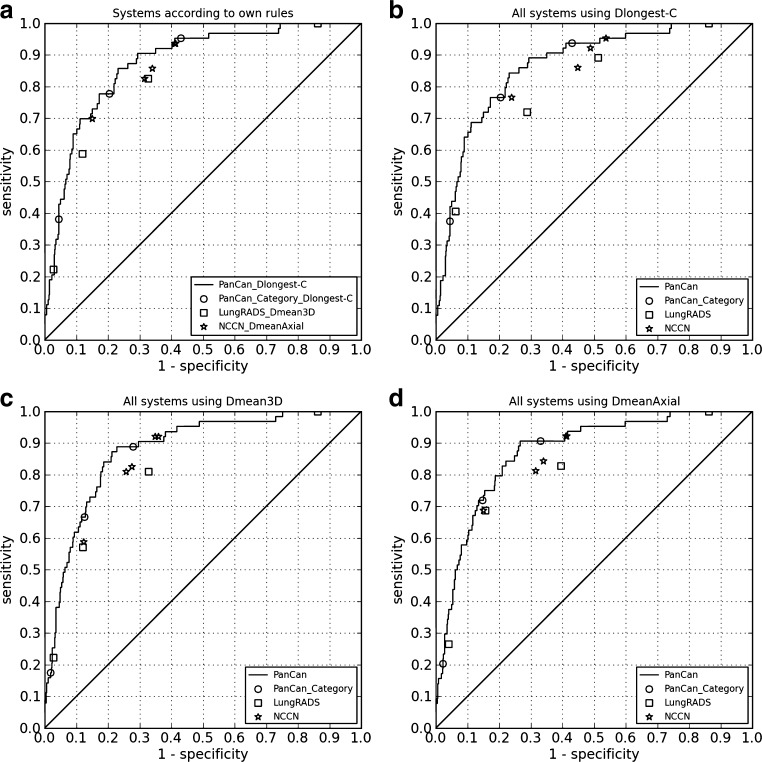
Performances of the PanCan model, Lung-RADS and the NCCN guidelines are illustrated in ROC curves. In all figures, the PanCan model is shown as a continuous curve based on the continuous nodule risk indexes and as operating points based on the categories for nodule risk index scores, similar to the Lung-RADS and NCCN categories. (**A**) All systems are visualized using their own nodule size diameter definitions, with D^longest-C^ for the PanCan model; (**B**) All systems are visualized using D^longest-C^ as nodule size definition; (**C**) All systems are visualized using D^mean3D^ as nodule size definition; **D**: All systems are visualized using D^meanAxial^ as nodule size definition

The PanCan model yielded a lower performance when using D^longest-M^ (0.869 vs. 0.874; p = 0.586); the difference was not statistically significant. Results are summarized in Table 3 and Fig. 4A.

**Table 3:** Performance comparisons between the PanCan model, Lung-RADS and the NCCN guidelines when using different nodule size definitions

Nodule size definition	PanCan	Lung-RADS	NCCN	PanCan vs. Lung-RADS	PanCan vs. NCCN	Lung-RADS vs. NCCN
Nodule size definition as published	0.874 ^1^	0.813^2^	0.836^3^	**p** = **0.003**	**p** = **0.010**	p = 0.175
	0.869 ^4^					
D^longest-C^	0.874	0.796	0.806	**p** < **0.001**	**p** < **0.001**	p = 0.507
D^mean3D^	0.880	0.813	0.845	**p** < **0.001**	**p** = **0.016**	p = 0.024
D^meanAxial^	0.879	0.812	0.836	**p** = **0.001**	**p** = **0.003**	p = 0.139

^1^ Use of the longest diameter measured on axial sections derived from semi-automated volumetric segmentations

^2^ Use of the mean diameter based on 3D volumetric information, derived from semi-automated volumetric segmentations

^3^ Use of the mean of longest and perpendicular diameter measured on axial sections derived from semi-automated volumetric segmentations

^4^ Use of the longest diameter manually measured on axial sections

*D* ^*longest*-*C*^ longest diameter on axial sections, derived from computerized semi-automated segmentations, *D* ^*mean3D*^ mean diameter based on volumetric information, derived from computerized semi-automated segmentations, *D* ^*meanAxial*^ mean of longest and perpendicular diameter on axial sections, derived from computerized semi-automated segmentations

Statistically significant differences are defined at p < 0.017, and indicated in bold

### Impact of nodule size definition on performance per system

All systems yielded the highest AUC-values when using D^mean3D^ for nodule size, followed by D^meanAxial^, and the worst performance was achieved when using D^longest-C^. Differences for the PanCan model (AUC 0.880, 0.879, 0.874) and Lung-RADS (0.813, 0.812, 0.796) were not statistically significant.

The NCCN guidelines (0.845, 0.836 and 0.806) yielded a statistically equivalent AUC of 0.845 when using D^mean3D^, and 0.836 using D^meanAxial^. However, using D^longest-C^ the AUC was 0.806 being significantly inferior to D^mean3D^ (p = 0.005) and D^meanAxial^ (p = 0.016). See Table 4.

**Table 4:** Impact of nodule size definition on performance of the PanCan model, Lung-RADS and the NCCN guidelines, expressed in AUC values

Nodule size definition	PanCan	Lung-RADS	NCCN
D^longest-C^ vs. D^mean3D^	0.874 vs. 0.880 p = 0.217	0.796 vs. 0.813 p = 0.190	0.806 vs. 0.845 **p** = **0.005**
D^mean3D^ vs. D^meanAxial^	0.880 vs. 0.879 p = 0.781	0.813 vs. 0.812 p = 0.914	0.845 vs. 0.836 p = 0.450
D^longest-C^ vs. D^meanAxial^	0.874 vs. 0.879 p = 0.140	0.796 vs. 0.812 p = 0.169	0.806 vs. 0.836 **p** = **0.016**

*D* ^*longest*-*C*^ longest diameter on axial sections, derived from computerized semi-automated segmentations, *D* ^*mean3D*^ mean diameter based on volumetric information, derived from computerized semi-automated segmentations, *D* ^*meanAxial*^ mean of longest and perpendicular diameter on axial sections, derived from computerized semi-automated segmentations

Statistically significant differences are defined at p < 0.017, and indicated in bold

### Impact of nodule size definition on performance between systems

Using the D^meanAxial^ for all systems yielded a statistically significant superiority of the PanCan model to Lung-RADS (AUC 0.879 vs. 0.812; p = 0.001) and the NCCN guidelines (0.879 vs. 0.836; p = 0.003).

Applying the D^longest-C^ resulted in a substantial performance drop of Lung-RADS (0.796) and NCCN (0.806), making both systems significantly inferior to PanCan (0.874; both p <0.001).

The use of D^mean3D^ yielded again a statistically significant superiority of the PanCan model to Lung-RADS (0.880 vs. 0.813; p <0.001), and the NCCN guidelines (0.880 vs. 0.845; p <0.016).

No statistically significant differences occurred between Lung-RADS and NCCN with p = 0.507, p = 0.024 and p = 0.139, for D^longest-C^, D^mean3D^ and D^meanAxial^, respectively. Results are summarized in Table 3. Figures 4B, C and D illustrate the ROC curves of the PanCan model, and category operating points for Lung-RADS, NCCN guidelines and the PanCan model when uniformly applying the three nodule size definitions.

## Discussion

Accurate differentiation between high-risk nodules requiring more intense work-up and low-risk nodules is essential for the implementation of a cost-effective lung cancer screening programme. For decision support and standardization, various scoring systems have been developed, all using nodule size as the most important criterion. Nevertheless, they show differences with respect to the use of subject demographics, nodule morphological features, definitions of nodule size and cut-off points. The purpose of this study was to assess performance differences between the PanCan model, Lung-RADS and the NCCN guidelines for the differentiation between malignant and benign screen-detected nodules using baseline information only.

Our results suggest that the three scoring systems indeed show significant performance differences; the PanCan model outperformed the NCCN guidelines and Lung-RADS. This significant superiority of the PanCan model was seen when applying the individual system's nodule size definitions and when applying uniformly the same nodule size definition (D^longest-C^, D^mean3D^ or D^meanAxial^).

No statistically significant difference was seen between Lung-RADS and the NCCN guidelines, either when using the system's specific diameter definitions or when uniformly applying any of the nodule size definitions.

All three systems showed the poorest performance when using D^longest-C^, and the strongest performance when using the mean diameter obtained from semi-automated volumetric segmentations (D^mean3D^). This result suggests that taking the three-dimensional information into account is superior to two dimensions (e.g. axial measurements alone). Whether true volumetry versus mean diameter obtained from thee-dimensional information will result in a significant performance difference can only be speculated. Nevertheless, we expect that automatic or semi-automatic assessment of volumetry will help to decrease inter-observer variability.

However, in contrast to the PanCan model and Lung-RADS, the NCCN guidelines showed a statistically significant susceptibility towards the definition of the nodule size with a significant superiority of D^meanAxial^ and D^mean3D^ over D^longest-C^. D^mean3D^, the only diameter definition using the three-dimensional extent of the nodule, was not found to perform significantly different from D^meanAxial^ for any of the three systems. This is important because it means that diameter measurements can be done on axial scans alone without using other projection planes.

From these results we conclude that apart from nodule size thresholds, nodule size definition has also a significant impact on the performance of such risk estimation systems, and should therefore be defined and documented carefully.

In addition to nodule size and nodule type, nodule growth is an important malignancy predictor, and the main rationale behind the acquisition of follow-up studies [12–14]. Growth is considered in the two categorical systems, but not in the PanCan model that is designed for risk estimation of lesions detected on baseline screening CTs. In order to be able to compare the categorical strategies with the PanCan model, we excluded this criterion. However, by doing so the potentials of the two categorical systems were restricted.

We know that manual diameter measurements are prone to substantial observer variability [15]. Using manual diameter measurements (D^longest-M^) yielded a lower performance for the PanCan model compared to the semi-automated diameter measurements (D^longest-C^), but the difference did not reach significance.

In this study, the PanCan model achieved a lower performance for nodules derived from the DLCST compared to the validation dataset from the BCCA (0.881 vs. 0.970) [6]. This difference is likely to be caused by different inclusion criteria between the datasets. The PanCan study and the BCCA dataset included smaller nodules than the DLCST, resulting in a lower prevalence of small benign nodules in our study (median size 5 mm, range 3–90 mm) compared to the BCCA dataset (median size 3 mm, range 1–29 mm) [6]. Furthermore, PFNs and calcified nodules were excluded in the PanCan model, but were annotated in the DLCST. To account for these design differences, both calcified and perifissural nodules were given a nodule risk index of 0% for the PanCan model in our study, which occurred in 151 nodules (151/740, 20%). Another contributor to the lower performance of the PanCan model in the current dataset is the fact that McWilliams et al. computed the performance of the model on a nodule level. However, we determined the performance of the PanCan model on participant level using the risk-dominant nodules, which introduced a selection bias towards larger benign nodules that are more difficult to discriminate from malignant lesions. We did so to more closely approximate the clinical setting, in which a person-specific analysis is preferred over a nodule-specific analysis.

Other nodule management systems have been published [16, 17] that have similar categories and criteria to the models discussed here, but differ in details. For this study, we decided to include these three widely known systems. However, as discussed by Baldwin [18], this does not automatically translate into extensive implementation in clinical practice of these models and guidelines.

It has to be stated that our data analysis is confined to the performance of how well the PanCan category 4, Lung-RADS category 4A/4B and 1.2016 NCCN category 6 predict malignancy and thus select nodules that require immediate work-up or eventually will become malignant. We did not compare how well the various follow-up algorithms perform for nodules kept under surveillance.

Our study compares various models in a retrospective study set-up. With more widely applied screening, there is likely to be an increasing need for a large-scale prospective audit taking multiple risk models into account. This should also address the current debate whether to apply diameter measurements or volumetry for nodule growth assessment.

Our study has limitations of which the most important one refers to the use of ROC statistics to evaluate the performance of clinical decision rules. As pointed out by Perandini et al., referring to the validation study of four prediction rules, ROC analysis is designed to estimate the performance of a binary classifier system and to determine an optimal threshold value to be used as discriminator [19]. The risk estimation systems tested in our study, however, use either a continuous scale (the PanCan model) or multiple categorical thresholds (Lung-RADS and NCCN) to balance a nodule's risk to represent a malignancy and the likelihood of being benign. The different biological behavior of fast growing aggressive and slowly growing less aggressive malignancies further complicates the clinical decision making. In the PanCan study, 20% of the lung cancers did not develop from the largest (risk-dominant) nodule [6]. Nevertheless we decided to use ROC analysis as the most widely used method of assessing the accuracy of a diagnostic test. In addition, by having the pathological standard available with 9 years of follow-up, we were able to dichotomously divide our study nodules into benign and malignant, and, lastly, there is to our knowledge no statistical test at hand that would be more suited to evaluate the complex multifactorial decision making of managing screen- detected nodules.

Other limitations of our study include a relatively small number of lung cancers (65 in total) and methodological differences between the three scoring systems that we had to exclude in order to compare the three systems with each other. Firstly, we only considered one-time information and disregarded information on nodule growth which is part of Lung-RADS and the NCCN guidelines. Secondly, Lung-RADS offers an additional category that allows radiologists to upgrade a category 3 or 4 nodule to category 4X if visually accessible criteria are present, making the nodule more suspicious. This subjective procedure was disregarded in our study [4]. These issues may have contributed to a lower performance of Lung-RADS and the NCCN guidelines. In that respect our study points to the importance of prospective evaluation of the clinical impact of differences in recommendation between the PanCan model and Lung-RADS.

Furthermore, the NCCN guidelines have recently been updated (version 1.2017) and harmonized with Lung-RADS. Nevertheless, we decided to keep the results of the previous NCCN guidelines (version 1.2016) in this study to include the performance of a third diameter definition on risk estimation. Lastly, the NCCN guidelines included separate rules for multiple pure ground-glass nodules, based on their size and the presence of a dominant lesion. However, there is no clear definition of the dominant lesion, and up to now it has not been shown that the presence of multiple pure ground-glass nodules represents an increased risk factor [20]. Therefore, the presence of multiple (pure ground-glass) nodules was not taken as a separate risk factor in our study.

In conclusion, the PanCan model performs significantly better than Lung-RADS and the 1.2016 NCCN guidelines for differentiation between malignant and benign nodules, detected on baseline screening CT and without taking nodule growth into account. Different nodule size definitions have an impact on the performance of the three systems, with statistically significant influence only for the NCCN guidelines.

## Abbreviations and acronyms

ACR: American College of Radiology
AUC: Area under the curve
BCCA: British Columbia Cancer Agency
DLCST: Danish Lung Cancer Screening Trial
D^longest-C^: Longest diameter on axial sections, derived from computerized semi-automated segmentations
D^longest-M^: Longest diameter on axial sections, derived from manual measurements
D^mean3D^: Mean diameter based on volumetric information, derived from computerized semi-automated segmentations
D^meanAxial^: Mean of longest and perpendicular diameter on axial sections, derived from computerized semi-automated segmentations
LDCT: Low-dose computed tomography
NCCN: National Comprehensive Cancer Network
NELSON: Dutch-Belgian Lung Screening Trial
PanCan: Pan-Canadian Early Detection of Lung Cancer Study
ROC: Receiver operating characteristic

## References

[1] Cressman S, Lam S, Tammemagi MC (2014). Resource utilization and costs during the initial years of lung cancer screening with computed tomography in Canada. J Thorac Oncol.

[2] Pinsky PF, Gierada DS, Nath PH, Kazerooni E, Amorosa J (2013). National lung screening trial: variability in nodule detection rates in chest CT studies. Radiology.

[3] Singh S, Pinsky P, Fineberg NS (2011). Evaluation of reader variability in the interpretation of follow-up CT scans at lung cancer screening. Radiology.

[4] Lung-RADS Assessment Categories, Version 1.0. American College of Radiology. Lung CT Screening Reporting and Data System (Lung-RADS™) Web site. http://www.acr.org/Quality-Safety/Resources/LungRADS. Release date April 28, 2014. Accessed September 15, 2014

[5] National Comprehensive Cancer Network Guidelines, Version 1.2016, Lung Cancer Screening. Web site. Release date June 23, 2015. http://www.nccn.org/professionals/physician_gls/f_guidelines.asp#detection

[6] McWilliams A, Tammemagi MC, Mayo JR (2013). Probability of cancer in pulmonary nodules detected on first screening CT. N Engl J Med.

[7] Winkler Wille MM, van Riel SJ, Saghir Z (2015). Predictive accuracy of the PanCan lung cancer risk prediction model - external validation based on CT from the Danish lung cancer screening trial. Eur Radiol.

[8] Wille MM, Dirksen A, Ashraf H (2016). Results of the randomized Danish lung cancer screening trial with focus on high-risk profiling. Am J Respir Crit Care Med.

[9] Van Klaveren RJ, Oudkerk M, Prokop M (2009). Management of lung nodules detected by volume CT scanning. N Engl J Med.

[10] Tammemagi MC, Lam S (2014). Screening for lung cancer using low dose computed tomography. BMJ..

[11] DeLong ER, DeLong DM, Clarke-Pearson DL (1988). Comparing the areas under two or more correlated receiver operating characteristic curves: a nonparametric approach. Biometrics.

[12] Horeweg N, van Rosmalen J, Heuvelmans MA (2014). Lancet Oncol..

[13] Lee HJ, Goo JM, Lee CH, Yoo CG, Kim YT, Im JG (2007). Nodular ground-glass opacities on thin-section CT: size change during follow-up and pathological results. Korean J Radiol.

[14] Lee JH, Park CM, Lee SM, Kim H, McAdams HP, Goo JM (2016) Persistent pulmonary subsolid nodules with solid portions of 5 mm or smaller: Their natural course and predictors of interval growth. Eur Radiol 26(6):1529–1537 10.1007/s00330-015-4017-4 26385803

[15] van Riel SJ, Sanchez CI, Bankier AA (2015). Observer variability of classification of pulmonary nodules on low-dose CT imaging and its effect on nodule management. Radiology.

[16] Callister ME, Baldwin DR, Akram AR (2015). British thoracic society guidelines for the investigation and management of pulmonary nodules. Thorax.

[17] Gould MK, Donington J, Lynch WR (2013). Evaluation of individuals with pulmonary nodules: when is it lung cancer? Diagnosis and management of lung cancer, 3rd ed: American College of Chest Physicians evidence-based clinical practice guidelines. Chest.

[18] Baldwin DR (2015). Development of guidelines fort he management of pulmonary nodules: toward better implmentation. Chest.

[19] Perandini S, Soardi GA, Motton M, Critique of Al-Ameri MS (2015). Risk of malignancy in pulmonary nodules: A validation study of four prediction models. Lung Cancer.

[20] Yankelevitz DF, Yip R, Smith JP (2015). CT screening for lung cancer: nonsolid nodules in baseline and annual repeat rounds. Radiology.

